# Presence and molecular characterization of *Cryptosporidium* and *Giardia* in recreational lake water in Tianjin, China: a preliminary study

**DOI:** 10.1038/s41598-018-20902-3

**Published:** 2018-02-05

**Authors:** Shumin Xiao, Yan Zhang, Xiaoyun Zhao, Liping Sun, Sike Hu

**Affiliations:** 1 School of Environmental and Municipal Engineering, Tianjin Chengjian University, Tianjin, 300384 P.R. China; 2 Tianjin Key Laboratory of Aquatic Science and Technology, Tianjin, 300384 P.R. China; 3 School of Medicine, Nankai University, Tianjin, 300071 P.R. China

## Abstract

Little is known about the occurrence of *Cryptosporidium* and *Giardia* in recreational water in China. A total of 52 samples were collected from recreational lakes in Tianjin during a high-occurrence season (June–October) for the waterborne cryptosporidiosis and giardiasis, and the occurrence and genotypes of *Cryptosporidium* and *Giardia* were investigated. The results showed that 82.7% (43) and 98.1% (51) of samples were positive for *Cryptosporidium* oocyst and *Giardia* cysts, respectively. The mean concentration of parasites was 3.65 oocysts/10 L and 12.58 cysts/10 L, respectively. Molecular characterization revealed that the presence of *Cryptosporidium parvum*, *C. andersoni*, *C. hominis*, *C. meleagridis*, *C. fragile*, *C. ubiquitum*, and *Giardia lamblia* assemblage A, B and D. The protozoan contamination in the studied lakes may originate from animal feces on ground, which was washed into the lake by stormwater runoff. Nevertheless, there is a potential risk of infection during recreational activities in the lake because the dominant detected protozoan genotypes are common human pathogens. Moreover, microbial indicators analysis does not adequately indicate the protozoan contamination in recreational water. The information from this study will be valuable for future protozoan source tracking, and any further control interventions against *Cryptosporidium* and/or *Giardia* infection associated with recreational water.

## Introduction

Recreational water has been well documented through outbreaks and epidemiologic studies as a transmission vehicle for pathogens^1^. Because of their high resistance to the environment and their high infectivity, *Cryptosporidium* and *Giardia* are the key etiological agents of waterborne disease^2,3^. At least 176 recreational water-associated outbreaks attributed to the two parasites have been documented in the five-year period between January 2011 and December 2016 in developed countries, while no outbreak is reported in developing countries^2^. The distortion reflecting the global pattern of distribution may be resulted from the substantial improvement in data reporting and the establishment of surveillance systems in developed countries^4^. In fact, it is well known that the highest infection prevalence of parasitic protozoa occurred in developing countries, due to their low economic status and poor sanitation^5^. The public health implications of the environmental transport of *Cryptosporidium* and *Giardia* in these countries, consequently, should be estimated considering that both agents are transmitted by the fecal-oral route and have caused foodborne and waterborne outbreaks.

In the most populous developing country, China, the existence of human cryptosporidiosis and giardiasis has been confirmed by a number of epidemiological investigations^6–8^, but only one cryptosporidiosis outbreak, which occurred in a pediatric hospital, has so far been reported^2,9^. This could be a picture of underreporting, as neither of the parasites is listed as a routine inspection item for diarrhea cases in the country. In spite of this, *Cryptosporidium* and *Giardia* have been widely reported in various animals, including wildlife, zoo animals, laboratory animals, farm animals, and house pets^10–15^. On the other hand, both protozoa have also been found in urban wastewater^16^, surface drinking water sources^17–19^ and recently in treated recreational waters (i.e. swimming pool waters)^20^. However, little is known about their occurrence in untreated recreational water. Additionally, more and more people in China are involved in water-related activities. Therefore, it is urgent for the protection of public health to investigate the occurrence of *Cryptosporidium* and *Giardia* in recreational water.

In addition, the species/genotype of the protozoa is another important factor that affects the outbreak of cryptosporidiosis and giardiasis, which has been often overlooked in the health risk assessment of *Cryptosporidium* and *Giardia* in environmental waters. In effect, not all *Cryptosporidium* and *Giardia* species can infect human^21–24^. Furthermore, the most common causative agents for cryptosporidiosis are *C. hominis*, *C. parvum*, *C. ubiquitum*, and *C. meleagridis*, though nearly 20 *Cryptosporidium* species and genotypes have been reported in humans^21,22^. Likewise, the only known *Giardia* species that causes human giardiasis is *Giardia lamblia*, in which subgroups of assemblages A and B are the main etiologies of diarrhea^23,24^. Consequently, understanding the species or assemblage of the parasites in environmental water is necessary to identify public health risk.

**Figure 1:**
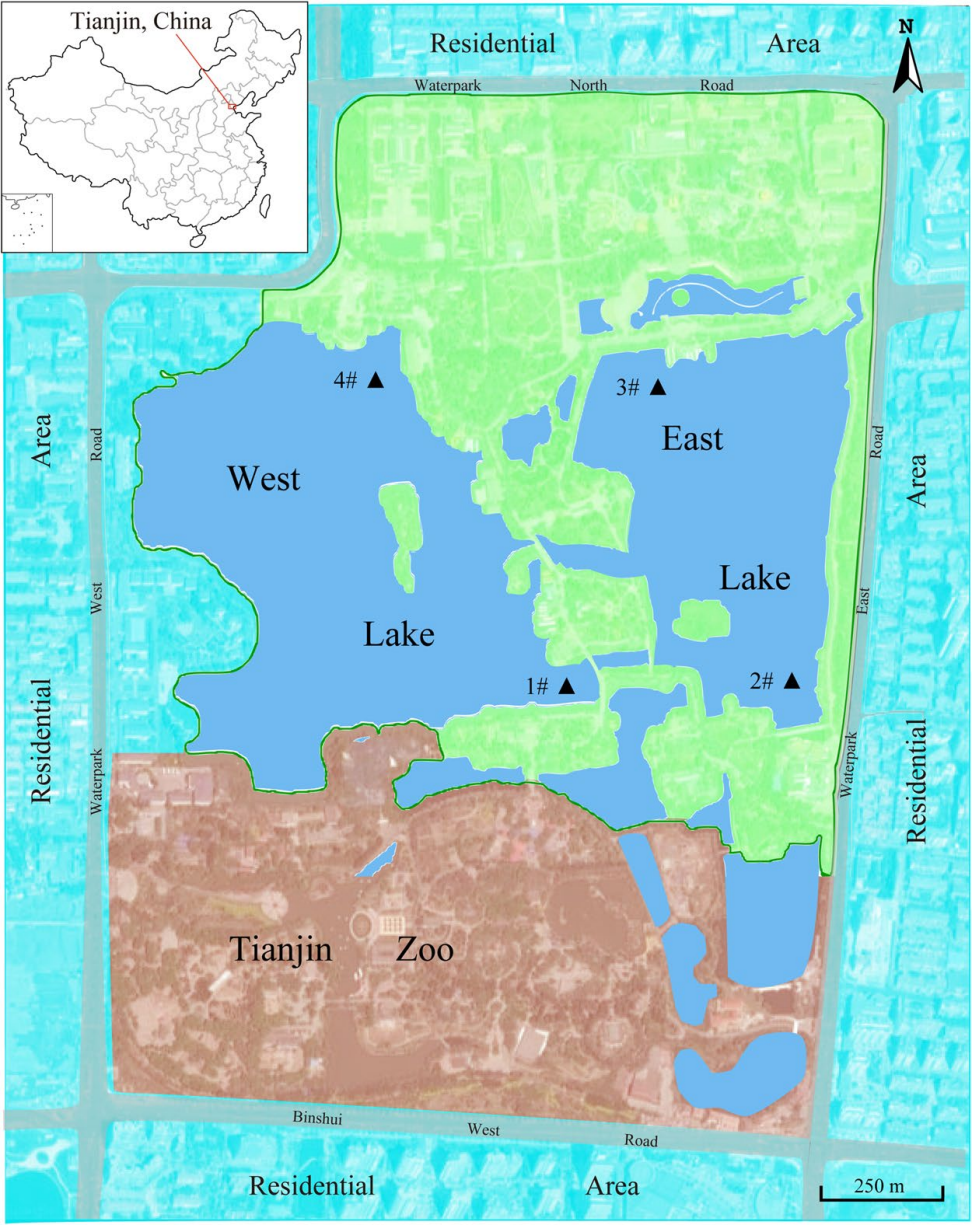
Recreational sampling sites (▲) in Tianjin Waterpark (Arabic numbers represent the sampling sites). The map was created by the authors using software of Adobe Illustrator CS4 (version 14.0.0, http://www.adobe.com/cn/products/illustrator.html).

This study was conducted to evaluate the presence and molecular characterization of *Cryptosporidium* and *Giardia* in untreated recreational water in the largest urban park of Tianjin, China (Fig. 1), during the high-occurrence season for the waterborne cryptosporidiosis and giardiasis^1,2,25–27^. The relationships between protozoan concentration and basic water quality indicator such as total coliforms, turbidity and pH, were also explored. The present preliminary survey is believed to be the first attempt to simultaneously detect and genotype the oocyst of *Cryptosporidium* and cyst of *Giardia* in recreational water in China, and will be helpful for the identification of public health risk and taking appropriate preventative measures.

## Results

### Precision of the method

The recovery efficiencies from the four replicates using the method involved filtration, flotation, labeling with monoclonal antibody, and microscopy, ranged between 32.50% and 50.83%, with a mean of 41.25%, for *Cryptosporidium* oocyst, whereas the average recovery rate for *Giardia* cyst was 38.32%, ranged from 27.01% to 47.45% (Table S1).

### Prevalence of *Cryptosporidium* and *Giardia* in water samples

Of the 52 tested recreational lake water samples, 43 (82.7%) were positive for *Cryptosporidium*, with 92.3% (12/13), 84.6% (11/13), 84.6% (11/13), and 69.2% (9/13) in samples from sites 1#, 2#, 3#, and 4#, respectively. *Giardia* cysts were detected in all samples (98.1%, 51/52) except for the one collected on October 5, 2015 from site 4#. The counts of parasites ranged from 0 to 15 oocysts with a mean of 3.65 oocysts, and 0 to 43 cysts with an average of 12.58 cysts per 10 liters (Table 1). The positive rate for both parasites in samples collected from sampling site 4# was lower than those from other sites. Nevertheless, the difference of concentration of *Cryptosporidium* oocyst or *Giardia* cyst among all of the sampling sites was not significant (P > 0.05).

**Table 1:** Occurrence of *Cryptosporidium* oocyst and *Giardia* cyst in water samples collected from recreational lakes^a^.

Sampling sites	No. of sample	*Cryptosporidium* (no. of oocysts/10 L)				*Giardia* (no. of cysts/10 L)			
		No. of positive (%)	Mean ± SD	Min. − Max.	95% UCL	No. of positive (%)	Mean ± SD	Min. − Max.	95% UCL
1#	13	12 (92.3%)	3.54 ± 2.85	(0–8)	5.08	13 (100%)	14.54 ± 7.57	(3–24)	18.57
2#	13	11 (84.6%)	3.85 ± 4.20	(0–15)	6.36	13 (100%)	12.23 ± 10.26	(3–43)	18.60
3#	13	11 (84.6%)	3.46 ± 3.64	(0–12)	5.56	13 (100%)	12.38 ± 8.82	(2–30)	17.25
4#	13	9 (69.2%)	3.77 ± 3.77	(0–12)	5.90	12 (92.3%)	11.15 ± 7.01	(0–27)	15.10
Total	52	43 (82.7%)	3.65 ± 3.54	(0–15)	4.63	51 (98.1%)	12.58 ± 8.35	(0–43)	14.90

^a^SD, standard deviation; UCL, upper confidence limit which was calculated based on 10,000 bootstrap samples using PASW statistics 18 software.

**Figure 2:**
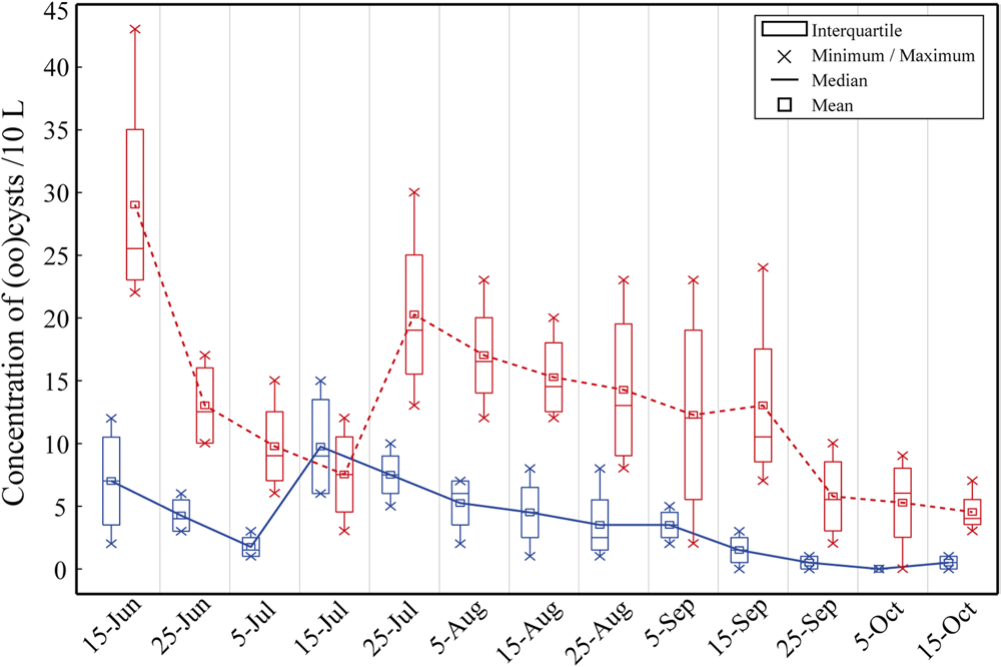
*Cryptosporidium* oocyst (blue continuous line) and *Giardia* cyst (red dashed line) concentrations in water samples collected from recreational lakes between June and October, 2015.

Variations of the concentrations of *Cryptosporidium* oocyst and *Giardia* cyst in different times are shown in Fig. 2. Except the peak in late July, the average counts of (oo)cysts in water samples showed a general downtrend during the survey.

### Genotyping of protozoa

Nested PCR amplifications were performed for *Cryptosporidium* and *Giardia*, respectively, on all the water samples and the expected size of amplicons were produced in 25% (13 out of 52) water samples. DNA sequencing of PCR products confirmed the presence of *C. parvum*, *C. andersoni*, *C. fragile*, *C. ubiquitum* and *Giardia* assemblage D in samples collected from site 1#; *C. parvum, C. andersoni*, *C. meleagridis*, *C. hominis* and *Giardia* assemblage A from site 2#; *C. andersoni* and *Giardia* assemblage A from site 3#; and *Giardia* assemblage B from site 4#. *Cryptosporidium parvum*, *C. andersoni*, and *Giardia* assemblage A were the most commonly detected protozoan species/assemblage (Table 2).

**Table 2:** Distribution of *Cryptosporidium* species and *Giardia* assemblage in water samples collected from recreational lakes.

**Sample sites**	**Species of** ***Cryptosporidium*** **(no. of samples)**						**Assemblage of** ***Giardia lamblia*** **(no. of samples)**		
	*C. parvum*	*C. andersoni*	*C. hominis*	*C. meleagridis*	*C. ubiquitum*	*C. fragile*	A	B	D
1#	2	1			1	1			1
2#	1	1	1	1			2		
3#		1					1		
4#								1	
All sites	3	3	1	1	1	1	3	1	1

### Correlation between protozoa and basic water quality parameters

The results of mean, median and range of the total coliforms, turbidity and pH of lake water are presented in Table 3. As expected, all of the samples were positive for total coliforms, with concentrations ranging between 2 × 10^3^ and 6.5 × 10^5^ most probable numbers (MPN) per liter. Nonparametric Spearman’s correlation two-tailed test showed there was a significant positive correlation between *Cryptosporidium* and *Giardia* as well as between them and turbidity (P < 0.01). Neither of the parasites displayed significant correlations with the total coliforms, which are commonly used to indicate microbial contamination (Table 4).

**Table 3:** Total coliforms, turbidity and pH of water samples collected from recreational lakes.

**Sample sites**	**Statistics**	**Total coliforms (×10** ^**3**^ **MPN/L)**	**Turbidity (NTU)**	**pH**
1#	Mean ± SD	66.87 ± 72.18	46.09 ± 25.45	8.87 ± 0.43
	Median	60	38	8.81
	Range	5–290	19–106	8.27–9.87
2#	Mean ± SD	81.47 ± 133.06	52.35 ± 19.36	8.86 ± 0.39
	Median	40	500	8.76
	Range	2–530	28–99	8.44–9.76
3#	Mean ± SD	73.04 ± 147.74	54.18 ± 26.03	8.86 ± 0.41
	Median	22	47	8.78
	Range	6–590	31–129	8.41–9.82
4#	Mean ± SD	93.47 ± 159.1	50.26 ± 31.82	8.84 ± 0.39
	Median	54	41	8.79
	Range	7–650	22–134	8.24–9.83
All sites	Mean ± SD	78.71 ± 129.32	50.72 ± 25.59	8.86 ± 0.39
	Median	41	43	8.79
	Range	2–650	19–134	8.24–9.87

**Table 4:** Correlations between the concentration of parasitic pathogens, total coliforms, turbidity and pH of the water analyzed^a^.

	*Cryptosporidium*	*Giardia*	Total coliforms	Turbidity	pH
*Cryptosporidium*	—	0.669**	0.179	0.383**	−0.054
*Giardia*	0.669**	—	0.303	0.363**	0.134
Total coliforms	0.179	0.303	—	0.176	0.208
Turbidity	0.383**	0.363**	−0.176	—	−0.574**
pH	−0.054	0.134	0.208	−0.574**	—

^a^Spearman’s correlation coefficient (two-tailed test), ^*^P < 0.05, ^**^P < 0.01.

## Discussion

Currently, the monitoring of *Cryptosporidium* oocyst and *Giardia* cyst in water is largely performed by using the United States Environmental Protection Agency (USEPA) Method 1623, which involved filtration, isolation of (oo)cysts by immunomagnetic separation, immunofluorescence assay^28^. However, the high cost of Method 1623 has restricted its usage in most developing countries. A recent review found that only 5% of publications on *Cryptosporidium* and *Giardia* detection in water from Central/South America and Africa adopted the Method 1623^28^. Similar testing methods from the United Kingdom and other countries have been involved as alternatives^19^. In this study, we employed a concentration and purification method adopted by Japan^29^. As shown in Table S1, this method permitted mean recoveries of 41.25% (relative standard deviation (RSD), 21.54%) for *Cryptosporidium* and 38.32% (RSD, 26.09%) for *Giardia*, which could meet the acceptable levels of the Method 1623 (38–100% (RSD < 37%) for *Cryptosporidium*, 27–100% (RSD < 39%) for *Giardia*)^30^.

Oocysts of *Cryptosporidium* and cysts of *Giardia* have been reported in recreational water in many previous studies. In Spain, the concentration of *Cryptosporidium* in recreational rivers varied from 10 to 600 oocysts/10 L and *Giardia* ranged between 10 and 1600 cysts/10 L^31^. Each of them was much higher than that reported in the present study (3.46–3.85 oocysts/10 L and 11.15–14.54 cysts/10 L, respectively), while slightly lower protozoan contamination (1–4 *Cryptosporidium* oocysts and 1–8 *Giardia* cysts per 10 liters) was reported in some recreational lake waters from the Netherlands^32^. No *Cryptosporidium* oocyst was found in samples from a Malaysia recreational lake but a density of 1.7–11 cysts per 10 liters for *Giardia* was detected^33^. So the contamination of *Cryptosporidium* oocysts and *Giardia* cysts in the recreational lakes herein is within the range those reported elsewhere.

The occurrence of protozoa in environmental water samples is influenced by many factors, including the timing, frequency and site of sampling^34^. Sampling once in the dry season and the rainy season, respectively, was adopted in many previous studies^35,36^. However, given that summer and early autumn are the high-occurrence season for the waterborne cryptosporidiosis and giardiasis^1,2,25–27^, and there was no water recreation in the park in winter, the present study therefore focused on the presence of *Cryptosporidium* oocyst and *Giardia* cyst in the hot season. Due to their small size, slow settling velocity, and resistance to environmental stress, oocyst and cyst contamination can persist in water for a long time^27^. Additionally, the lake water was disturbed by boating and the sampling intervals were relatively short. These factors resulted in a high detection rate of parasites for all samples and no significant differences in protozoan positive rate or densities at different sampling sites.

Genotyping demonstrates that a total of 6 species of *Cryptosporidium* and 3 assemblages of *Giardia lamblia* present in this study (Table 2). Most of them have been well reported in animals across China, such as *C. parvum* in many kinds of animals^13^, *C. meleagridis* in chicken^37^, *C. ubiquitum* in deer and sheep^38^, *C. andersoni* in cattle^12,13^, and *Giardia lamblia* in dogs, cats and cattle^10,11^. Nevertheless, it is noteworthy that all the detected protozoan species/assemblages except *C. fragile* have been reported in humans^21,23,39^, and the dominant genotypes, i.e. *C. parvum*, *C. andersoni*, and *Giardia* assemblage A, are the common human pathogens in China^8,16^, showing their potential public threat and requiring the attention of public health authorities.

Several studies have shown that main sources for protozoan contamination of surface water identified were the intrusion of animal feces or wastewater due to heavy rains^40–42^. In this study, the replenishment patterns of the lakes depend mainly on tap water and occasional heavy rainfall, so the discharge of storm water may be the major contribution of parasitic pathogens contamination. This is consistent with the variations of concentrations of *Cryptosporidium* oocyst and *Giardia* cyst. That is, the downtrend of concentration over time suggesting that no continuous protozoa was discharged into the lake, whereas the peak in late July may be attributed to the wash from fecal-polluted ground by the heavy rainfall on 19 July, 2015 (http://tj.weather.com.cn, Fig. S1). Indeed, the number of oocyst and cyst shed by infected animals was believed to be as high as 10^6^–10^8^ per gram of feces^27,43^. Considering that all detected *Cryptosporidium* species and *Giardia* assemblages have been found in animals, particularly *C. fragile* has only been found in amphibians, the protozoa in this study appears to be derived from animals and may be associated to the nearby zoo since there are many animals including amphibians. Thus, it is one of the important questions for future studies to determine the source of protozoa in the lakes.

Physical-chemical properties such as turbidity and bacteria such as total coliforms are commonly used as indicators for monitoring water quality. Significant correlations were observed between turbidity and parasites, and between parasites themselves in the present study. These findings are in agreement with previous studies observed in a recreational river in Taiwan, China^44^, in the Three Gorges Reservoir in China^36^, and in a reservoir in Spain^35^. Nevertheless, no parasite was related to indicator bacteria in this study. In contrast, Graczyk, *et al*.^45^ found that bacteria count was a good indicator for the presence of *Giardia* and *Cryptosporidium* in marine recreational beach water. Meanwhile, they noted that water sample should be collected during times when bather numbers are high and tested in time. The strong resistance of *Cryptosporidium* and *Giardia* to environmental stress and long-term viability may be the main cause of different results of the above studies^46^. Previous study also found that fecal bacterial indicator was not an appropriate index to monitor the presence of *Cryptosporidium* or *Giardia* in treated recreational water^20^. Consequently, the analysis of microbial indicators does not adequately characterize contamination of protozoa in recreational water, especially if the sampling is not timely.

In conclusion, *Cryptosporidium* and *Giardia* were commonly detected in the recreational lakes, and their concentration levels are similar to those reported elsewhere. The protozoan contamination may be attributed to heavy rains that wash ground-polluted feces into the lake, and was related to the nearby zoo animals. As the dominant detected protozoan genotypes are common human pathogens, their potential threat requires the attention of public health authorities. In addition, microbial indicators analysis does not adequately indicate the protozoan contamination in recreational water. Future studies should therefore include follow-up work designed to assess the occurrence of protozoa of feces from residents nearby and the zoo animal, allowing a full evaluation of its public health.

## Material and Methods

### Study site

The studied lakes are located in the Tianjin Waterpark (39.080° to 39.095° N, 117.159° to 117.174°E), which is the largest comprehensive park within the city boundaries of Tianjin, China. All the lakes are connected together and the surface area is about 750,000 square meters. Apart from the surface runoff during storm, the lakes depend mainly on tap water for its replenishment. It is an aquatic venue used for rowing, interactive fountain, wading, and other water recreation activities. There were hundreds of people, mainly the elderly residents nearby, swim in the early morning and evening in summer, even though swimming is banned here.

### Sample collection and processing

During the period between June and October, which was the high-occurrence season for waterborne cryptosporidiosis and giardiasis^26,27,47,48^, water samples were collected from 4 sampling sties (as shown in Fig. 1) at ten-day intervals and a total of 52 samples were obtained. The sampling site 1# is close to the zoo in the south of the West lake and site 4# in the north, site 2# is located in the swimming area in the East lake and site 3# is near the cruise ship terminal. Each sample was collected using a 20-L plastic container. After collection, the samples were transported on ice to the laboratory immediately and parasitic pathogens in the samples were concentrated by a membrane filter dissolution method described previously^49^. Briefly, water samples were filtered and then the mixed cellulose ester membrane filter (diameter, 142 mm; pore size, 1 μM; Advantec MFS, Inc. Japan) was dissolved in acetone solutions, followed by centrifugation at 1,050 × g for 10 min at 4 °C. The packed pellets were resuspended in a suitable volume (2–10 mL) of distilled water. Half of the resuspension from each sample was used for protozoan enumeration and the other half for DNA extraction.

### Morphological examination and enumeration for protozoa

The recovered *Cryptosporidium* oocysts and/or *Giardia* cysts in the pellet were separated from debris by flotation on Percoll-sucrose gradients (specific gravity, 1.10)^49^. Each purified sample was stained with 100 μL of combined fluorescein isothiocyanate (FITC) conjugated anti-*Cryptosporidium* and anti-*Giardia* monoclonal antibodies (Waterborne, Inc., New Orleans, LA) in a humid dark chamber at room temperature for 30 min, and then stained with 50 μL of 4′,6′-diamidino-2-phenylindole (DAPI, Waterborne) solution (0.4 μg/mL) for 10 min. The prepared slides were subsequently examined microscopically at 400× magnification using fluorescence microscopy (Olympus, Japan) for the detection and enumeration of *Cryptosporidium* oocysts and *Giardia* cysts according the USEPA Method 1623^30^.

To evaluate the recovery efficiency of the method used, the initial precision and recovery efficiency was determined by spiking four 10 L of purified water samples with suspension containing enumerated oocysts and cysts^50^. Furthermore, a negative control assay, in which all the procedures were performed using deionized water instead of the water sample, was performed on each batch of samples to determine if any contamination occurred during the analysis. All negative control analyzes were negative for oocysts or cysts.

### Genotyping of *Cryptosporidium* and *Giardia*

Genomic DNA was extracted from each of the sample concentrates using the FastDNA SPIN kit for soil (MP Biomedicals, France) and eluted into 50 μL of reagent-grade water according to previous study^51^. Small subunit (SSU) rRNA-based nested PCR followed by sequencing techniques were employed to determine the species/genotypes of the protozoa according to previous study^52^. Briefly, a 435-bp fragment of *Cryptosporidium* SSU rRNA locus and a 292-bp fragment of *Giardia* SSU rRNA gene were amplified by two nested PCR, respectively. The sequence of all primers and PCR conditions are specified in Table S2. All positive secondary PCR products were sent to Beijing Augct Co., Ltd. for direct sequencing with the secondary PCR primers using ABI 3730 automated DNA sequencer (BigDye Terminator Chemistry). Nucleotide sequences obtained in the study were aligned with reference SSU rRNA sequences downloaded from the Genbank using the Clustal W programs and analyzed to determine *Cryptosporidium* species/genotypes and *Giardia* assemblages using phylogenetic trees.

### Microbiological analysis, turbidity and pH measurement

According to the *Chinese standard examination method for drinking water-microbiological parameters*, five-tube most probable numbers (MPN) procedure was used to enumerate total coliforms^53^. Briefly, after ten-fold serial dilution, 1.0 mL of each serial dilution was transferred to five tubes of lactose peptone broth containing inverted Durham tubes, and then incubated at 37 °C for 24 ± 2 h. All positive presumptive tubes that demonstrated an acidic reaction or gas production were submitted to the confirmed phase with total coliform test by using eosin methylene blue agar medium^53^. The turbidity and pH were measured on site for each sample with portable photometer.

### Statistical analysis

The paired-samples *t*-test was employed to evaluate the relationship between the concentrations of *Cryptosporidium* oocyst and *Giardia* cyst. The association between total coliform, turbidity, pH and protozoan concentrations was correlated using the nonparametric Spearman’s correlation two-tailed test. Differences with P values of <0.05 were defined as being statistically significant. All statistical tests were performed using PASW Statistics 18 computer software package.

### Data availability

All relevant data are fully available without restriction.

## Electronic supplementary material

Supplemental Material
