# Supplementary material for: Presence and molecular characterization of Cryptosporidium and Giardia in recreational lake water in Tianjin, China: a preliminary study
Source: Sci Rep. 2018 Feb 5;8:2353. doi: 10.1038/s41598-018-20902-3 (PMC5799358; doi:10.1038/s41598-018-20902-3)
Supplement: Supplementary file 1 — Supplemental Material [file 41598_2018_20902_MOESM1_ESM.pdf]

# Supplemental Material for

## "Presence and molecular characterization of *Cryptosporidium* and *Giardia* in recreational lake water in Tianjin, China: a preliminary study"

**Shumin Xiao [1,2,*], Yan Zhang [1], Xiaoyun Zhao [1], Liping Sun [1], Sike Hu [3,*]**

[1] School of Environmental and Municipal Engineering, Tianjin Chengjian University, Tianjin 300384, PR China

[2] Tianjin Key Laboratory of Aquatic Science and Technology, Tianjin 300384, PR China

[3] School of Medicine, Nankai University, Tianjin 300071, PR China

* **Correspondence**: xiaoshumin@tcu.edu.cn (S.X.), Tel.: +86-22-23085117; huskie@126.com (S.H.)

| | |
|---|---|
| Pages | S1–S4 |
| Figure | S1 |
| Table | S1–S2 |

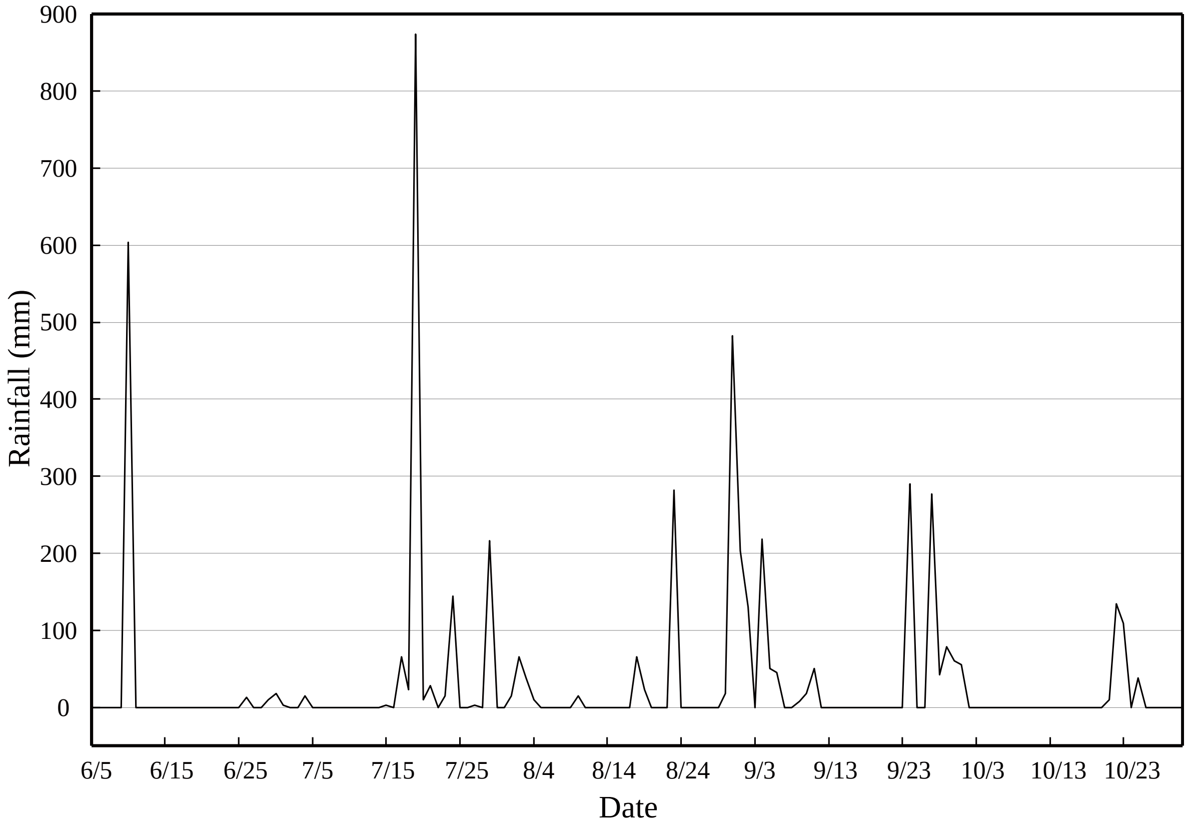


**Figure S1** Daily rainfalls in Tianjin, China from the beginning of June to the end of October, 2015.

**Table S1**. Initial precision and recovery (IPR) test of *Cryptosporidium* oocysts and *Giardia* cysts for the method used.

| **Protozoa** | **Spiked number** | **Recovered number (n=4)** | **Recovery (range) (%)** | **Relative standard deviation (%)** |
|---|---|---|---|---|
| *Cryptosporidium* oocyst | 120 | 49.5 (56, 39, 42, 61) | 41.25 (32.50–50.83) | 21.54 |
| *Giardia* cyst | 137 | 52.5 (63, 37, 45, 65) | 38.32 (27.01–47.45) | 26.09 |

**Table S2**. Primers and conditions of the PCR reaction used in this study for amplification of *Cryptosporidium* and *Giardia* at SSU rRNA gene loci.

| Parasite | PCR | Primers | PCR Parameters |
|---|---|---|---|
| *Crypto.* | 1st | CPB-DIAGF:5′-AAGCTCGTAGTTGGATTTCTG-3′<br>CPB-DIAGR:5′-TAAGGTGCTGAAGGAGTAAGG-3′ | 7 min at 94 °C followed by 35 cycles of 30 s at 94 °C , 60 s at 68 °C and 30 s at 72 °C |
| | 2nd | N-DIAGF2:5′-CAATTGGAGGGCAAGTCTGGTGCCAGC-3′<br>N-DIAGR2:5′-CCTTCCTATGTCTGGACCTGGTGAGT-3′ | 7 min at 94 °C followed by 35 cycles of 30 s at 94 °C , 60 s at 60 °C and 30 s at 72 °C |
| *Giardia* | 1st | GiaF:5′-AAGTGTGGTGCAGACGGACTC-3′<br>GiaR:5′-CTGCTGCCGTCCTTGGATGT-3′ | 5 min at 94 °C followed by 35 cycles of 45 s at 96 °C , 30 s at 55 °C and 45 s at 72 °C |
| | 2nd | RH11:5′-CATCCGGTCGATCCTGCC-3′<br>RH4:5′-AGTCGAACCCTGATTCTCCGCCAGG-3′ | 3 min at 96 °C followed by 35 cycles of 45 s at 96 °C , 30 s at 59 °C and 30 s at 72 °C |
